# Pressure-Dependent Confinement Effect of Ionic Liquids in Porous Silica

**DOI:** 10.3390/nano9040620

**Published:** 2019-04-16

**Authors:** Teng-Hui Wang, En-Yu Lin, Hai-Chou Chang

**Affiliations:** Department of Chemistry, National Dong Hwa University, Shoufeng, Hualien 974, Taiwan; 810712101@gms.ndhu.edu.tw (T.-H.W.); 410312036@gms.ndhu.edu.tw (E.-Y.L.)

**Keywords:** ionic liquid, IR spectroscopy, silica, high pressure

## Abstract

The effect of confining ionic liquids (ILs) such as 1-ethyl-3-methylimidazolium tetrafluoroborate [C_2_C_1_Im][BF_4_] or 1-butyl-3-methylimidazolium tetrafluoroborate [C_4_C_1_Im][BF_4_] in silica matrices was investigated by high-pressure IR spectroscopy. The samples were prepared via the sol-gel method, and the pressure-dependent changes in the C–H absorption bands were investigated. No appreciable changes were observed in the spectral features when the ILs were confined in silica matrices under ambient pressure. That is, the infrared measurements obtained under ambient pressure were not sufficient to detect the interfacial interactions between the ILs and the porous silica. However, dramatic differences were observed in the spectral features of [C_2_C_1_Im][BF_4_] and [C_4_C_1_Im][BF_4_] in silica matrices under the conditions of high pressures. The surfaces of porous silica appeared to weaken the cation-anion interactions caused by pressure-enhanced interfacial IL-silica interactions. This confinement effect under high pressures was less obvious for [C_4_C_1_Im][BF_4_]. The size of the cations appeared to play a prominent role in the IL-silica systems.

## 1. Introduction

Porous silica, synthesized using established procedures such as the sol-gel method, have a high surface area and are used in many applications [1,2,3,4,5,6]. The high surface area property makes porous silica promising candidates for the development of ionogels, biological applications, and the fabrication of mechanical systems [1]. The concept of ionogels is associated with ionic liquids immobilized by solid-like matrices (porous silica, for example) [2,3,4,5,6]. The precursors of porous silica are usually silicon alkoxides such as tetramethyl orthosilicate (TMOS) and tetraethylorthosilicate (TEOS) [5,6].

Ionic liquids (ILs) are salts whose melt temperature is less than 100 ºC because of the difficulty of stacking asymmetric and bulky cations and anions [7,8,9,10,11,12,13,14,15,16,17]. Due to the non-volatility and liquid state at room temperature, ILs have been used in energy storage devices, carbon dioxide absorptions, and dye-sensitized solar cells [7,8,9,10,12]. Typically, imidazolium-based ionic liquids are most extensively studied, and the cations are characterized by an imidazolium polar head and alkyl tail [7,8,9,10]. Aggregation of cations and anions in bulk ILs via Coulombic forces and hydrogen bonding has been proposed, where the cation-anion interactions lead to heterogeneous or organized cluster structures in the IL [7,8,9,10].

The confinement effect is a phenomenon wherein molecules or ions are trapped in molecular-dimension rooms (caves in porous materials), where their interactions with the pore-wall surfaces may change the physicochemical behaviors of the molecules or ions [1,8]. The molecules or ions entrapped in porous networks may show physical properties dissimilar to those of the neat liquid states, and the surface interactions with the pores may disturb the molecular or ionic associations [1,8]. As ILs are confined in an inorganic matrix, the mixtures form a two-phase structure, where the solid and liquid phases interconnect throughout the mixture. Much effort has been made to probe the changes in the properties of the ILs, upon physical confinement. These have led to the ILs in immobilized forms, owing to the solid porous matrix [1,2,3,4,5,6] being applied in a wide variety of applications. Some studies have shown that ILs entrapped in silica matrices (ionogels) have unique properties as compared to the neat ILs [3,4,5,6]. The physicochemical nature of the ionogel is determined by the interplay of the cation-anion associations and IL-surface interactions. The confinement of ILs in the host matrix leads to a partial disruption of the cation-anion interactions and cluster structures and cause ion–matrix interactions, resulting in changes to the phase-transition temperatures [3,4,5,6]. The confinement effect may also relax the crystallization rate of ILs. In addition to porous silica, some authors proposed that polyvinylidene fluoride (PVDF) and the matrix made from amine hardener and an epoxy prepolymer could act as the host network [11]. Gelled electrolyte-containing ILs may have applications in the future of lithium-ion batteries [12]. Understanding the interfacial structures of ILs at solid surfaces is crucial for extending the application of ILs in energy-storage devices. However, hysteretic anion-cation exchanges on a solid surface (in the first ionic layer) makes it demanding to understand what happens if various surfaces are interfaced with ILs [18,19].

The application of high pressure is an excellent technique to investigate the ordering of ILs on a solid surface [20,21,22]. The changes in the spectral characteristics induced by high pressures are of particular interest. The local structures of ILs appear disturbed under high pressure, and pressure-enhanced interfacial interactions may occur between the ILs and the solid surfaces, under high pressures. The various degrees of associations between the ILs and the solid surfaces at high pressures may arise from a reorganization of local structures and the hydrogen bonding network [20,21,22,23]. Upon compression, the relative weights of the intermolecular forces defining the aggregation states and the intramolecular interactions (molecular bonding) are changed. To obtain a further understanding of the pressure-dependence of the confinement effect [24], we applied high pressure to study the local structures of the ILs confined in porous silica. We note that P > 30 GPa is needed to change the electronic structures of the samples. Thus, the pressure used in this study (approximately 2 GPa) mainly affect local structures and interionic distances. Infrared spectroscopy is sensitive to monitor the local structures and potential energy environments, although the crystal and glass conformations may not be conclusively determined by IR. Thus, high-pressure IR spectroscopy may offer a specific means to detect the local structures of ionic liquids in confined geometries.

## 2. Materials and Methods

1-Ethyl-3-methylimidazolium tetrafluoroborate ([C_2_C_1_Im][BF_4_], 97%, Sigma-Aldrich, St. Louis, MO, USA), 1-butyl-3-methylimidazolium tetrafluoroborate ([C_4_C_1_Im][BF_4_], ≥97.0%, Sigma-Aldrich), tetraethylorthosilicate (TEOS, 99.999%, Sigma-Aldrich), and formic acid (FA, 98–100%, Merck-KGaA, Darmstadt, Germany) were used to prepare the samples. The ionogels (IL/porous silica) were synthesized according to the template method [5,6]. Formic acid and TEOS were mixed in a molar ratio of 8:1, followed by the addition of the IL ([C_2_C_1_Im][BF_4_] or [C_4_C_1_Im][BF_4_]) at 5 wt%. The mixture was allowed to gellify for approximately one week. Afterward, the ethanol, ethyl formate, and the remaining FA were removed by vacuum for 24 h. The powder-like samples were dried at 180 ºC using a moisture analyzer (MS-70, A&D Company, Tokyo, Japan).

Fourier transformed infrared spectra of the samples were collected on an IR spectrophotometer (Spectrum RXI, Perkin-Elmer, Naperville, IL, USA) equipped with a lithium tantalite (LITA) detector and a 5× beam condenser. A DAC (diamond anvil cell) of the Merrill–Bassett design was used to generate pressures of up to approximately 2 GPa. For high-pressure infrared measurements, two type-IIa diamonds were used. The infrared spectra of empty DAC were measured first in order to remove the infrared absorption of the two diamond anvils. The samples were placed in a 0.3-mm-diameter hole in a metal gasket (thickness 0.25 mm) mounted on the diamond anvil cell. To avoid saturation of the infrared absorption bands, part of the sample-hole was filled with CaF_2_ crystals. For each high-pressure spectrum, 1000 scans were collected. The FTIR spectrometer was operated at data point resolution of 2 cm^−1^ (a resolution of 4 cm^−1^). The pressure calibration was carried out following Wong’s method [25,26]. The FTIR spectra of the ionogel samples at ambient pressure were obtained by the CaF_2_ pellet method.

## 3. Results and Discussion

**Figure 1:**
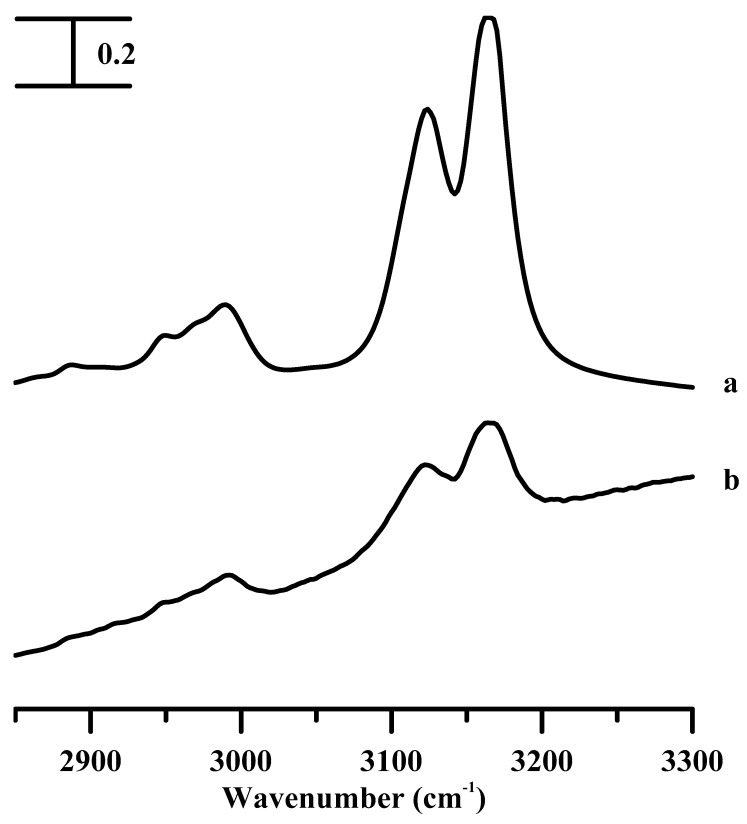
Infrared spectra of the (a) pure [C_2_C_1_Im][BF_4_] and (b) [C_2_C_1_Im][BF_4_] in a silica matrix, recorded at ambient pressure.

Figure 1 displays the IR spectra of the pure [C_2_C_1_Im][BF_4_] (curve a) and [C_2_C_1_Im][BF_4_] in a silica matrix (curve b) recorded under ambient pressure. The IR spectrum of neat [C_2_C_1_Im][BF_4_] in Figure 1a shows two demarcated peaks at 3124 and 3164 cm^−1^, corresponding to the C^2^–H and C^4,5^–H vibrations, respectively, of the aromatic imidazolium cation [27,28]. There exist three alkyl C–H bands located in the 2925–3025 cm^−1^ region as shown in Figure 1a. The absorption baseline in Figure 1b can be attributed to the Si–OH groups in the silica surface of the ionogel [20]. A comparison of the spectral absorptions in Figure 1b and Figure 1a showed no significant band-shift or feature-change for the C–H vibrational absorptions of the [C_2_C_1_Im][BF_4_] confined in the silica matrix at ambient pressure. The results in Figure 1 indicate that the vibrational spectroscopic measurements of the IL and ionogel, at ambient pressure, were not sufficient to distinguish the IL-silica interactions from the IL-IL associations.

**Figure 2:**
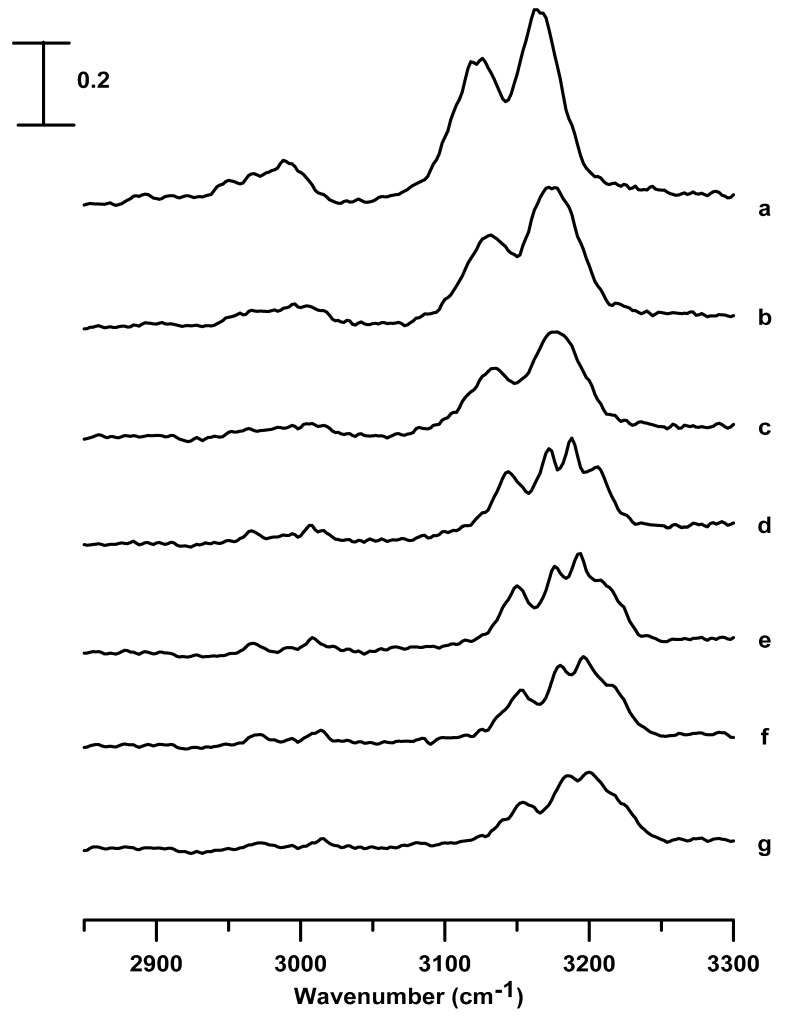
Infrared spectra of the pure [C_2_C_1_Im][BF_4_] obtained at (a) ambient pressure and at (b) 0.4, (c) 0.7, (d) 1.1, (e) 1.5, (f) 1.8, and (g) 2.5 GPa.

Figure 2 shows the IR spectra of neat [C_2_C_1_Im][BF_4_] obtained under ambient pressure (curve a) and at pressures of 0.4 (curve b), 0.7 (curve c), 1.1 (curve d), 1.5 (curve e), 1.8 (curve f), and 2.5 GPa (curve g). In the pressure range from ambient to 0.7 GPa (in Figure 2a–c), the peak broadens in width and blue-shifts in frequency are observed, upon compression, in the C^2^–H bands (at ~3124 cm^−1^) and C^4,5^–H bands (at ~3164 cm^−1^); these are blue-shifted to 3135 and 3177 cm^−1^, respectively, at a pressure of 0.7 GPa (Figure 2c). The alkyl C–H bands in the 2925 to 3025 cm^−1^ region also exhibit broadening and blue-shifts with an increase in pressure (≤0.7 GPa), as shown in Figure 2a–c. As the pressure is raised to 1.1 GPa, as in Figure 2d, the wavenumber of the C^2^–H stretching band increased to 3143 cm^−1^ with a decrease in the bandwidth. A phase transition or the formation of organized structures may occur, as shown in Figure 2d. As shown in Figure 2d, the C^4,5^–H band (at ~3177 cm^−1^) is split into three peaks at 3170, 3187, and 3206 cm^−1^ because of the pressure-enhanced interactions. That is, as the [C_2_C_1_Im][BF_4_] is compressed to 1.1 GPa, solid [C_2_C_1_Im][BF_4_] may be present in multiple stable local structures for the C^4,5^–H groups. The imidazolium C–H absorptions in the range between 3100 and 3200 cm^−1^ are complicated by the hydrogen bonding in a cluster mode and Fermi-resonance interactions [27,28]. Previous studies indicated that imidazolium C–H stretching modes of large cluster structures (hydrogen-bonding network) occurred at high wavenumbers [28]. Thus, we assigned the C^4,5^–H bands at 3170, 3187, and 3206 cm^−1^ in Figure 2d to the vibrations of isolated, medium, and large associated local structures, respectively, of the C^4,5^–H groups. The alkyl C–H bands located at ~2967 and 3007 cm^−1^ became dramatically sharp in bandwidth at 1.1 GPa, as shown in Figure 2d. The differences in the spectral absorptions of Figure 2c (0.7 GPa) and d (1.1 GPa) can be related to the generation of an anisotropic environment in Figure 2d, owing to the local alkyl C–H structure changes. It is known that hydrogen bonding leads to ring stacking of ILs and vibrational frequency shifts of imidazolium cations [7,8,9]. Our results in Figure 2 indicate the important roles played by micro-heterogeneity and hydrogen bonding in [C_2_C_1_Im][BF_4_]. As the pressure is increased to 1.5 GPa, the absorption intensity of the C^4,5^–H band at ~3209 cm^−1^ decreases slightly, accompanied by band broadening, as shown in Figure 2e. The decrease in the absorbance of the C^4,5^–H absorption at ~3209 cm^−1^ in Figure 2e may originate from the relaxation of the C^4,5^–H local structures of the pure [C_2_C_1_Im][BF_4_] high-pressure phases upon further compression. The C–H absorptions show continuous band broadening in Figure 2f,g.

**Figure 3:**
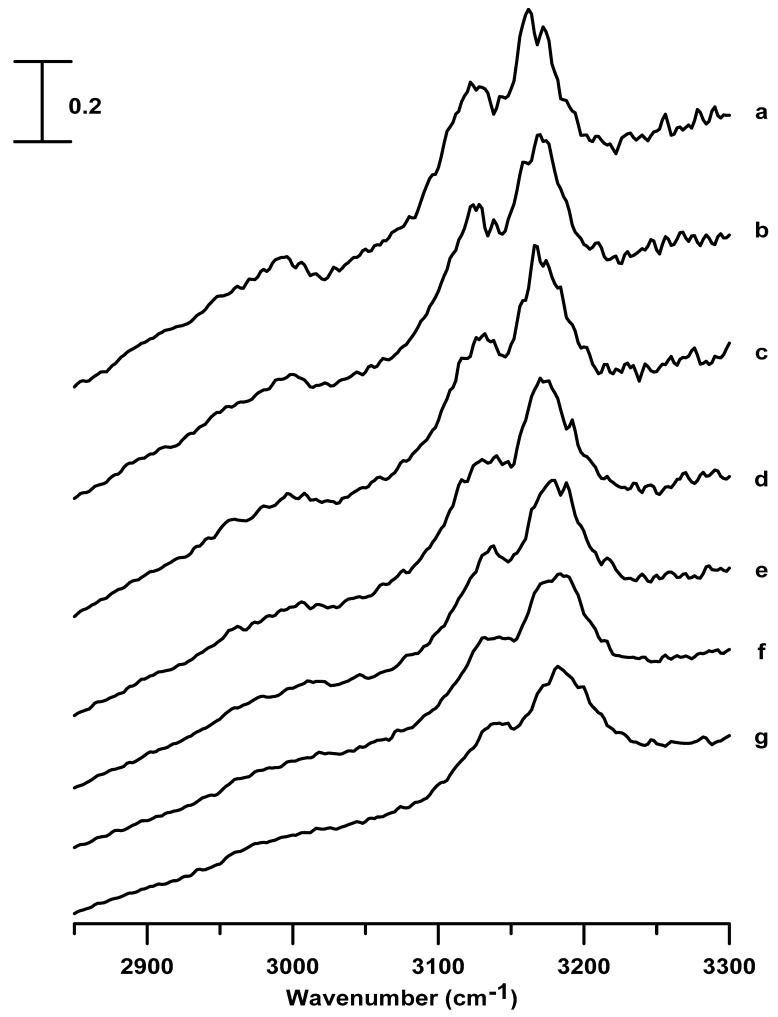
Infrared spectra of [C_2_C_1_Im][BF_4_] in a silica matrix obtained at (a) ambient pressure and at (b) 0.4, (c) 0.7, (d) 1.1, (e) 1.5, (f) 1.8, and (g) 2.5 GPa.

Figure 3 shows the IR spectra of [C_2_C_1_Im][BF_4_] in a silica matrix obtained under ambient pressure (curve a) and at pressures of 0.4 (curve b), 0.7 (curve c), 1.1 (curve d), 1.5 (curve e), 1.8 (curve f), and 2.5 GPa (curve g). The C^4,5^–H and C^2^–H absorptions show the blue-shifts in frequency to 3174 and 3128 cm^−1^, respectively, with subtle band broadening in Figure 3c during compression. The aliphatic C–H modes of the alkyl group absorptions display band broadening and a slight blue-shift in the frequency at the pressure of 0.7 GPa in Figure 3c. As the pressure is elevated to 1.1 GPa, the splitting of the C^4,5^–H absorptions is not observed in Figure 3d, unlike the case of the pure [C_2_C_1_Im][BF_4_], being split into three separate bands in Figure 2d. The C^4,5^–H of [C_2_C_1_Im][BF_4_] in a silica matrix shows monotonic blue-shifts in frequency and band-broadening in Figure 3d–g. The alkyl C–H bands in Figure 3d–g also show blue-shifts in frequency and band-broadening, in contrast to the sharp alkyl C-H absorptions in Figure 2d–g. The results in Figure 3d–g suggest that the IL-IL associations have changed as [C_2_C_1_Im][BF_4_] is confined in a silica matrix. The local structures of [C_2_C_1_Im][BF_4_], i.e., the multiple stable local structures, may be perturbed by the silica matrices via the porous surface-IL interactions. In other words, the porous silica may intervene in the organization of the [C_2_C_1_Im][BF_4_] at high pressures (1.1–2.5 GPa), as shown in Figure 3d–g.

**Figure 4:**
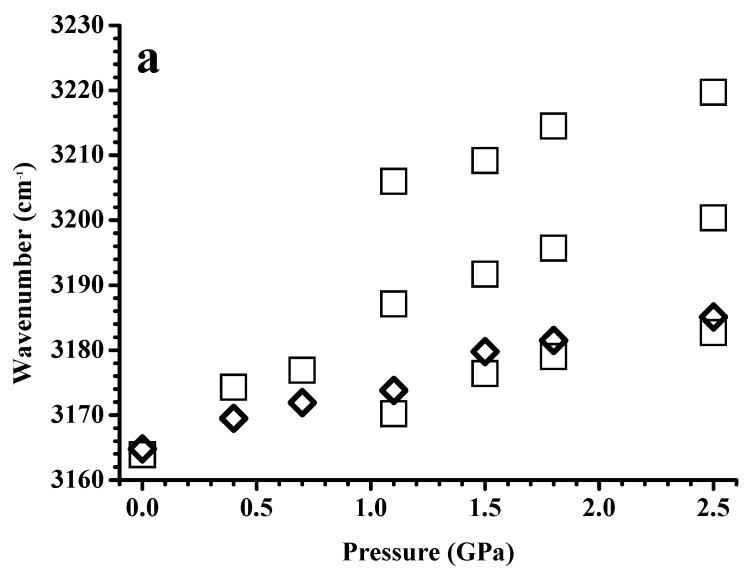

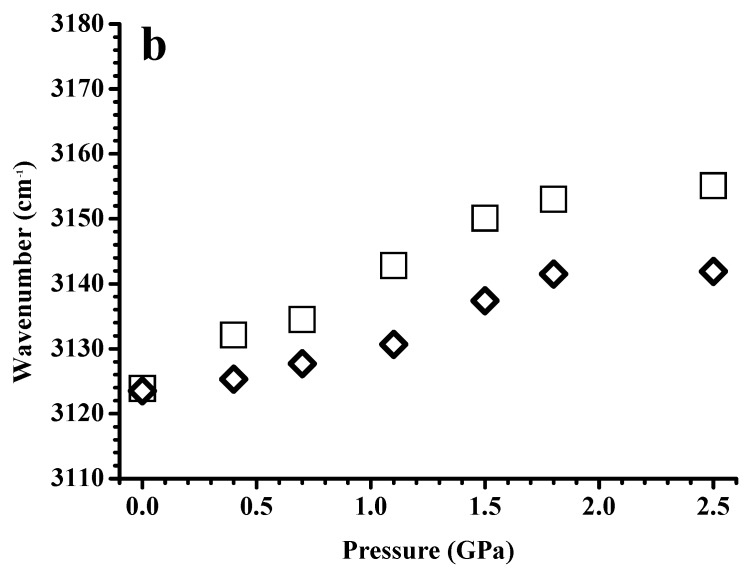
Pressure dependence of the C–H stretching frequencies at (**a**) 3164 and (**b**) 3124 cm^−1^ of the pure [C_2_C_1_Im][BF_4_] (squares) and [C_2_C_1_Im][BF_4_] in a silica matrix (diamonds).

Figure 4 shows the pressure-dependence of the band-shifts in frequency for imidazolium C–H absorptions of the neat [C_2_C_1_Im][BF_4_] and [C_2_C_1_Im][BF_4_] in a silica matrix. The stretching frequencies of the C^4,5^–H (Figure 4a) and C^2^–H (Figure 4b) bands at ambient pressure are almost identical for pure [C_2_C_1_Im][BF_4_] and [C_2_C_1_Im][BF_4_] in a silica matrix. At the pressures of 0.4 and 0.7 GPa, the C^4,5^–H (Figure 4a) and C^2^–H (Figure 4b) stretching modes undergo mild red-shifts in frequency upon adding the silica matrix. The red-shifts in frequency induced by the presence of the silica matrix became obvious for C^2^–H under high pressure (1.1–2.5 GPa), as shown in Figure 4b. Ludwig’s group reported that the imidazolium C^2^–H stretching band could be described by a major peak at ~3125 cm^−1^ associated with a minor absorption at 3104 cm^−1^, corresponding to the associated structure and isolated structure, respectively [28]. The absorption components at 3125 cm^−1^ and 3104 cm^−1^ may arise from the large clusters (associated structures) and small clusters (isolated structures), respectively. The decrease in bandwidth of the C^2^–H absorption in Figure 2d can be attributed to the decline in the molar ratio of the isolated/associated forms of pure [C_2_C_1_Im][BF_4_] at high pressures. The dramatic decrease in the intensity of the isolated form (shoulder) is not observed in Figure 3d for [C_2_C_1_Im][BF_4_] in the silica matrix under high pressure. Therefore, the red-shifts in frequency in Figure 4b induced under high pressures by the silica matrix for C^2^–H may originate from the partial switch of the associated form to the isolated form. These results indicate the prominent role of hydrogen bonding in [C_2_C_1_Im][BF_4_]/silica systems [22,27]. The presence of the silica matrix appears to force the IL to form another arrayed structure, causing the C^2^–H isolated structures to increase in intensity under high pressure. We note that the C^2^–H associated structures remain the dominant species even at high pressures. As shown in Figure 4a, the C^4,5^–H peak of pure [C_2_C_1_Im][BF_4_] at ~3164 cm^−1^ is blue-shifted upon compression at P ≤ 0.7 GPa, and is then split into three absorptions (~3206, 3187, and 3170 cm^−1^) at P ≥ 1.1 GPa, corresponding to the isolated, medium, and large structures, respectively. Nevertheless, the C^4,5^–H absorptions of [C_2_C_1_Im][BF_4_] in a silica matrix show monotonic blue-shifts in frequency upon compression, as observed in Figure 4a. The top peak positions of C^4,5^–H of [C_2_C_1_Im][BF_4_] in a silica matrix are almost identical to the position of the isolated structures of pure [C_2_C_1_Im][BF_4_]. Consistent with the results of C^2^–H, the C^4,5^–H band also reveals an increase in the absorption intensity of the isolated form under high pressures. We note that in contrast to C^2^–H, the isolated C^4,5^–H structure is the dominant local conformation for the [C_2_C_1_Im][BF_4_] confined in the silica matrix. According to the experimental results in the literature [5], confinement of [C_2_C_1_Im][BF_4_] may result in the decrease in the dimensionality of solidification of the [C_2_C_1_Im][BF_4_] from a three-dimensional to a one-dimensional structure. Compared to published results [5], our pressure-dependent behaviors observed in Figure 2, Figure 3 and Figure 4 seem to support the trend of reduced dimensionality for [C_2_C_1_Im][BF_4_] in a silica matrix.

To obtain a clear comprehension into the consequence of the cation size on the pressure-induced isolation/association for pure ILs and IL in a silica matrix, the vibrational spectra of pure [C_4_C_1_Im][BF_4_] and [C_4_C_1_Im][BF_4_] in a silica matrix are studied. Figure S1 (in the Supplementary Materials) shows the IR spectra of the pure [C_4_C_1_Im][BF_4_] (curve a), and [C_4_C_1_Im][BF_4_] in a silica matrix (curve b) recorded under ambient pressure. The IR spectrum of pure [C_4_C_1_Im][BF_4_] in Figure S1a shows two imidazolium C–H peaks at 3120 (C^2^–H vibrational bands) and 3163 cm^−1^ (C^4,5^–H vibrational bands), similar to those displayed for the pure [C_2_C_1_Im][BF_4_] in Figure 1a. Three demarcated alkyl C–H bands at 2967, 2939, and 2876 cm^−1^ are observed, caused by the longer alkyl side-chain of the imidazolium of the [C_4_C_1_Im][BF_4_] cation as shown in Figure S1a. Similar to the case in Figure 1, the results obtained at ambient pressure for [C_4_C_1_Im][BF_4_] and [C_4_C_1_Im][BF_4_] in a silica matrix in Figure S1 are not sensitive enough to study the isolated/associated forms of pure [C_4_C_1_Im][BF_4_] and [C_4_C_1_Im][BF_4_] in a silica matrix.

**Figure 5:**
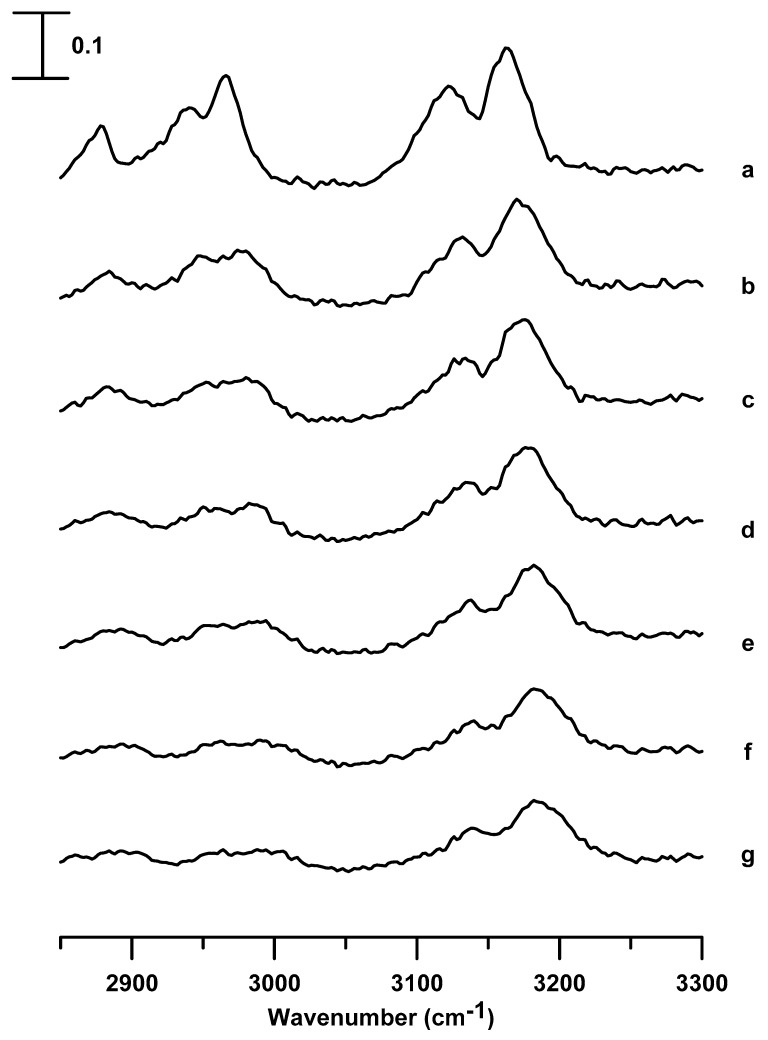
Infrared spectra of the pure [C_4_C_1_Im][BF_4_] obtained at (a) ambient pressure and at (b) 0.4, (c) 0.7, (d) 1.1, (e) 1.5, (f) 1.8, and (g) 2.5 GPa.

Figure 5 shows the IR spectra of the pure [C_4_C_1_Im][BF_4_] obtained under ambient pressure (curve a) and at pressures of 0.4 (curve b), 0.7 (curve c), 1.1 (curve d), 1.5 (curve e), 1.8 (curve f), and 2.5 GPa (curve g). As the pressure is increased (ambient–2.5 GPa), the C^2^–H (3120 cm^−1^), C^4,5^–H (3163 cm^−1^), and alkyl C–H (2967 cm^−1^) absorptions are blue-shifted in frequency accompanied by band broadening, in the case of the pure [C_4_C_1_Im][BF_4_], as shown in Figure 5. Upon compression to high pressures (ambient–2.5 GPa) in Figure 5, the C^4,5^–H band does not show the band-splitting, and the C^2^–H band does not reveal band-narrowing, as observed in Figure 2 for the pure [C_2_C_1_Im][BF_4_]. That is, the pure [C_4_C_1_Im][BF_4_] may form a less organized structure at high pressures. This phenomenon of forming a less organized structure, presented in Figure 5, is quite different from that of forming organized structures (in the case of pure [C_2_C_1_Im][BF_4_] in Figure 2), which can be attributed to the longer alkyl side-chains of the aromatic ring of the [C_4_C_1_Im][BF_4_] cation. That is, the possibility of the organization is more for symmetric cations such as [C_2_C_1_Im] than for asymmetrical structures, such as [C_4_C_1_Im].

**Figure 6:**
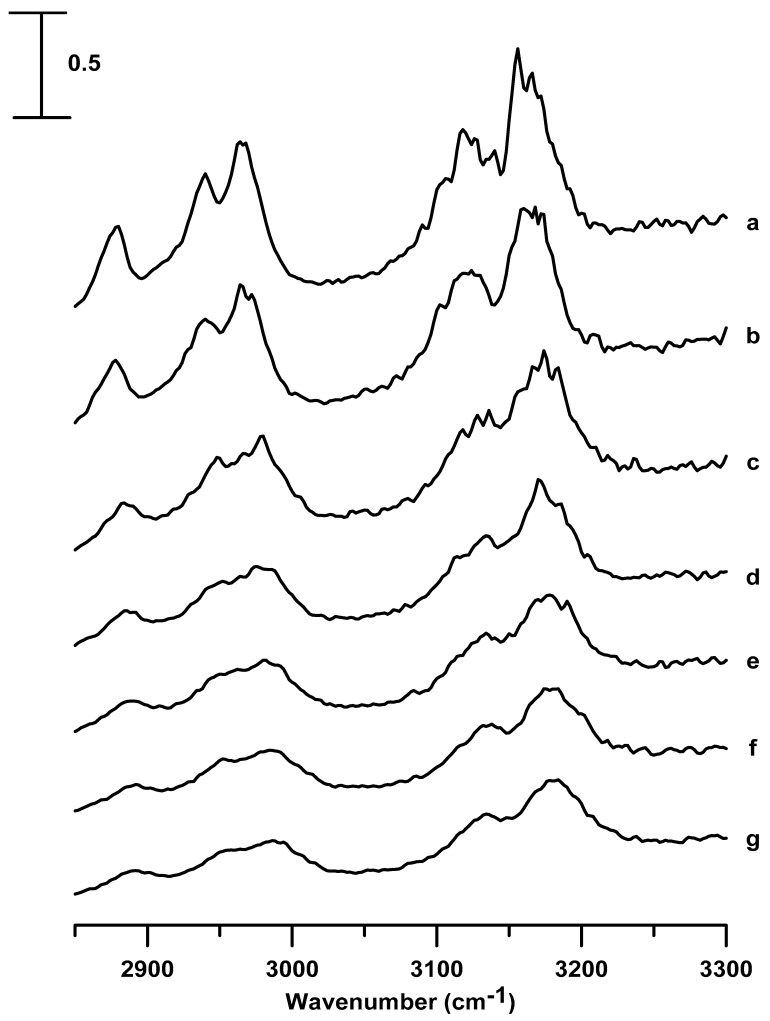
Infrared spectra of [C_4_C_1_Im][BF_4_] in a silica matrix obtained at (a) ambient pressure and at (b) 0.4, (c) 0.7, (d) 1.1, (e) 1.5, (f) 1.8, and (g) 2.5 GPa.

Figure 6 shows the IR absorptions of [C_4_C_1_Im][BF_4_] in a silica matrix obtained under ambient pressure (curve a) and at pressures of 0.4 (curve b), 0.7 (curve c), 1.1 (curve d), 1.5 (curve e), 1.8 (curve f), and 2.5 GPa (curve g). The spectral features of [C_4_C_1_Im][BF_4_] in a silica matrix, shown in Figure 6, are similar to those displayed for pure [C_4_C_1_Im][BF_4_] in Figure 5. In Figure 6, which presents the results of [C_4_C_1_Im][BF_4_] in a silica matrix, the C^4,5^–H (3164 cm^−1^), C^2^–H (3120 cm^−1^), and alkyl C–H (2967 cm^−1^) bands are blue-shifted accompanied by band broadening, as the pressure is surged from ambient to 2.5 GPa.

**Figure 7:**
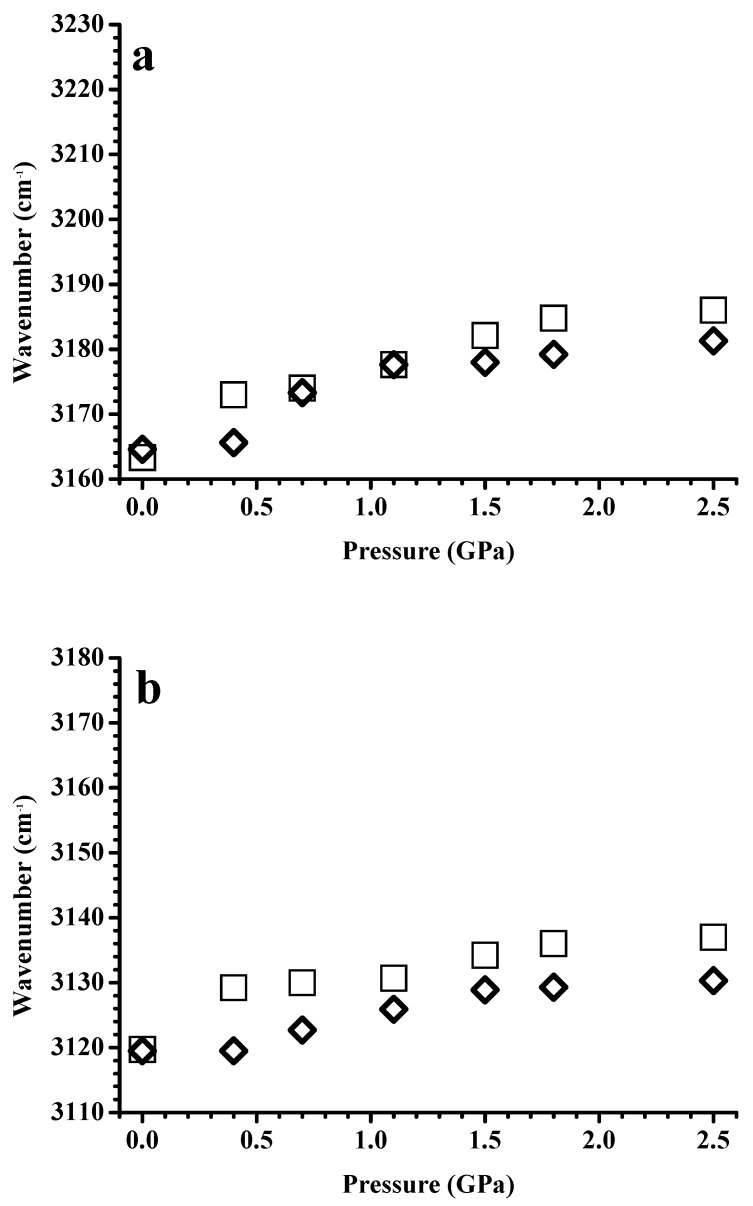
Pressure dependence of the C-H stretching frequencies at (**a**) 3163 and (**b**) 3120 cm^−1^ of the pure [C_4_C_1_Im][BF_4_] (squares) and [C_4_C_1_Im][BF_4_] in a silica matrix (diamonds).

Figure 7 shows the pressure-dependence of the band-shifts in frequencies for imidazolium C–H absorptions of pure [C_4_C_1_Im][BF_4_] and [C_4_C_1_Im][BF_4_] in a silica matrix. Figure S2 in the Supplementary Materials shows the pressure-dependence of the band-shifts of the alkyl C–H absorptions at ~2967 cm^−1^ for the pure [C_4_C_1_Im][BF_4_] and [C_4_C_1_Im][BF_4_] in a silica matrix. As shown in Figure 7 and Figure S2, remarkable blue-shifts in frequency occur upon compression to 0.4 GPa for pure [C_4_C_1_Im][BF_4_]. This observation suggests a phase transition of pure [C_4_C_1_Im][BF_4_] at 0.4 GPa. For [C_4_C_1_Im][BF_4_] in a silica matrix, initially, the C–H bands shift slightly in frequency (or show almost no change) at 0.4 GPa, but blue-shifts in frequency occur upon further increase of the pressure to 0.7 GPa, as shown in Figure 7 and Figure S2. These results indicate that the presence of the silica matrix may inhibit the solidification of [C_4_C_1_Im][BF_4_] via pressure-enhanced interfacial interactions. The C–H bands show mild frequency-shifts upon further compression for both pure [C_4_C_1_Im][BF_4_] and [C_4_C_1_Im][BF_4_] in a silica matrix. Minor differences are observed in the C–H vibrational frequency, between pure [C_4_C_1_Im][BF_4_] and [C_4_C_1_Im][BF_4_] in a silica matrix, under high pressures, as observed in Figure 7 and Figure S2. In other words, silica can be employed to change the hydrogen-bonded structure of [C_4_C_1_Im][BF_4_], as the pressure is increased. However, the changes in the aggregation behavior of [C_4_C_1_Im][BF_4_] via pressure-enhanced IL-surface interactions are less obvious than those of [C_2_C_1_Im][BF_4_].

To develop insight into the effect of anions, the preliminary pressure-dependent results of pure [C_4_C_1_Im][PF_6_] and [C_4_C_1_Im][PF_6_] in a silica matrix are displayed in Figures S3 and S4, respectively, in the Supplementary Materials. Figure S5 (in Supplementary Materials) shows pressure dependence of the C–H stretching frequencies at 3170 cm^−1^ of the pure [C_4_C_1_Im][PF_6_] and [C_4_C_1_Im][PF_6_] in a silica matrix, and the changes in aggregation behavior of [C_4_C_1_Im][PF_6_] caused by silica are revealed in Figure S5.

## 4. Conclusions

We conclude that ILs with short alkyl side-chains ([C_2_C_1_Im][BF_4_]) tended to form organized structures and that local C–H structures were affected significantly by the surface-IL interactions at high pressures. Both isolated and associated conformations existed in the pure [C_2_C_1_Im][BF_4_]. However, the associated form partially dissociated into isolated structures caused by pressure-enhanced IL-silica interfacial interactions. On the other hand, compared to [C_2_C_1_Im][BF_4_], the asymmetric [C_4_C_1_Im][BF_4_] could form a less organized solid, and the confinement effect induced by the silica matrix, upon compression, became less obvious for [C_4_C_1_Im][BF_4_] in a silica matrix. We note that evidence on the crystalline or glass-like state of ionic liquids can be offered by diffraction. Therefore, a sensitive detection technique such as diffraction may be helpful for future applications.

## Supplementary Materials

The following are available online at https://www.mdpi.com/2079-4991/9/4/620/s1, Figure S1: Infrared spectra of (a) pure [C_4_C_1_Im][BF_4_] and (b) [C_4_C_1_Im][BF_4_] in a silica matrix, recorded at ambient pressure, Figure S2: Pressure dependence of the C–H stretching frequencies at 2967 cm^−1^ of the pure [C_4_C_1_Im][BF_4_] (squares) and [C_4_C_1_Im][BF_4_] in a silica matrix (diamonds). Figure S3: Infrared spectra of the pure [C_4_C_1_Im][PF_6_] obtained at (a) ambient pressure and at (b) 0.4, (c) 0.7, (d) 1.1, (e) 1.5, (f) 1.8, (g) 2.5 GPa, and (h) back to ambient. Figure S4: Infrared spectra of [C_4_C_1_Im][PF_6_] in a silica matrix obtained at (a) ambient pressure and at (b) 0.4, (c) 0.7, (d) 1.1, (e) 1.5, (f) 1.8, and (g) 2.5 GPa. Figure S5: Pressure dependence of the C–H stretching frequencies at 3170 cm^−1^ of the pure [C_4_C_1_Im][PF_6_] (squares) and [C_4_C_1_Im][PF_6_] in a silica matrix (diamonds).
