# Supplementary material for: Pressure-Dependent Confinement Effect of Ionic Liquids in Porous Silica
Source: Nanomaterials (Basel). 2019 Apr 16;9(4):620. doi: 10.3390/nano9040620 (PMC6523140; doi:10.3390/nano9040620)
Supplement: Supplementary file 1 [file nanomaterials-09-00620-s001.pdf]

Supplementary Materials: Pressure-Dependent Confinement Effect of Ionic Liquids in Porous Silica

Commented [M1]: We did a layout for the supplementary according to our rules, please confirm

Teng-Hui Wang, En-Yu Lin and Hai-Chou Chang

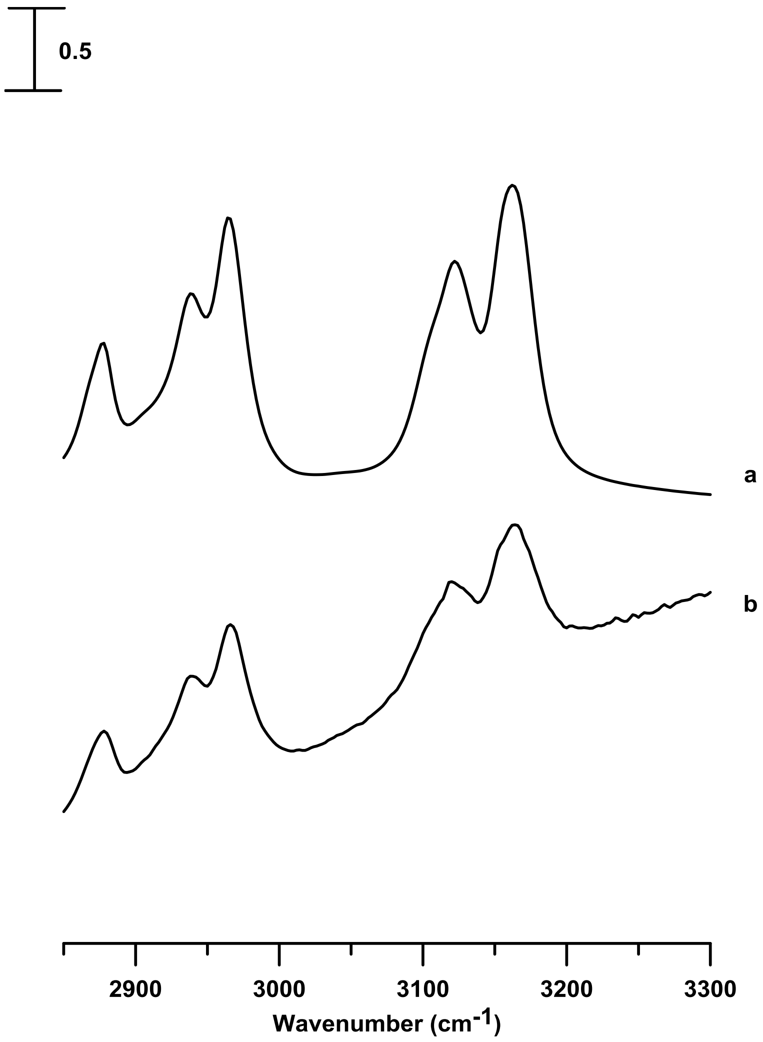

Figure S1. Infrared spectra of (a) pure [C4C1Im][BF4] and (b) [C4C1Im][BF4] in a silica matrix, recorded at ambient pressure.

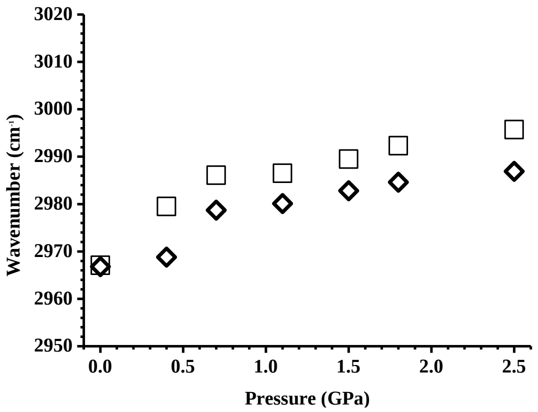

Figure S2. Pressure dependence of the C–H stretching frequencies at 2967 cm^{-1} of the pure [C4C1Im][BF4] (squares) and [C4C1Im][BF4] in a silica matrix (diamonds).

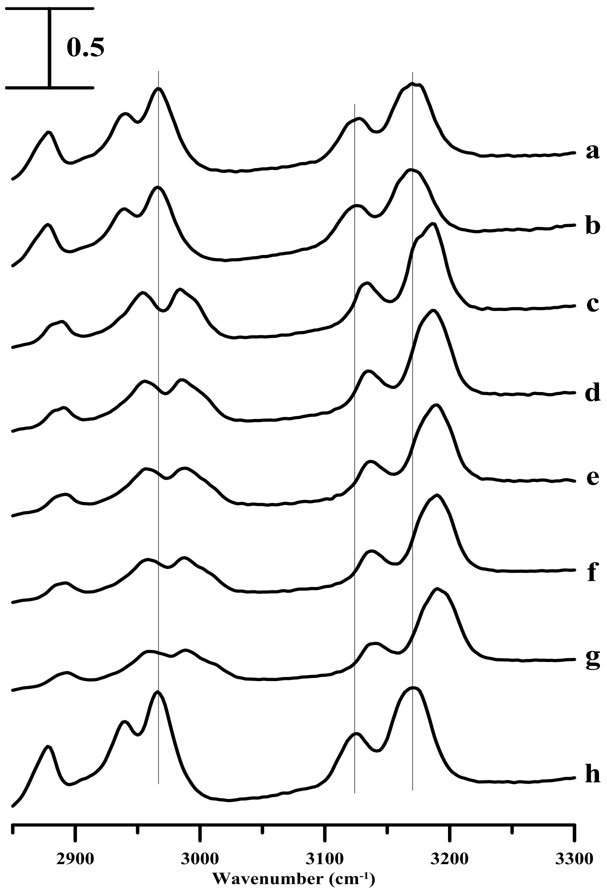

Figure S3. Infrared spectra of the pure [C4C1Im][PF6] obtained at (a) ambient pressure and at (b) 0.4, (c) 0.7, (d) 1.1, (e) 1.5, (f)1.8, (g) 2.5 GPa, and (h) back to ambient.

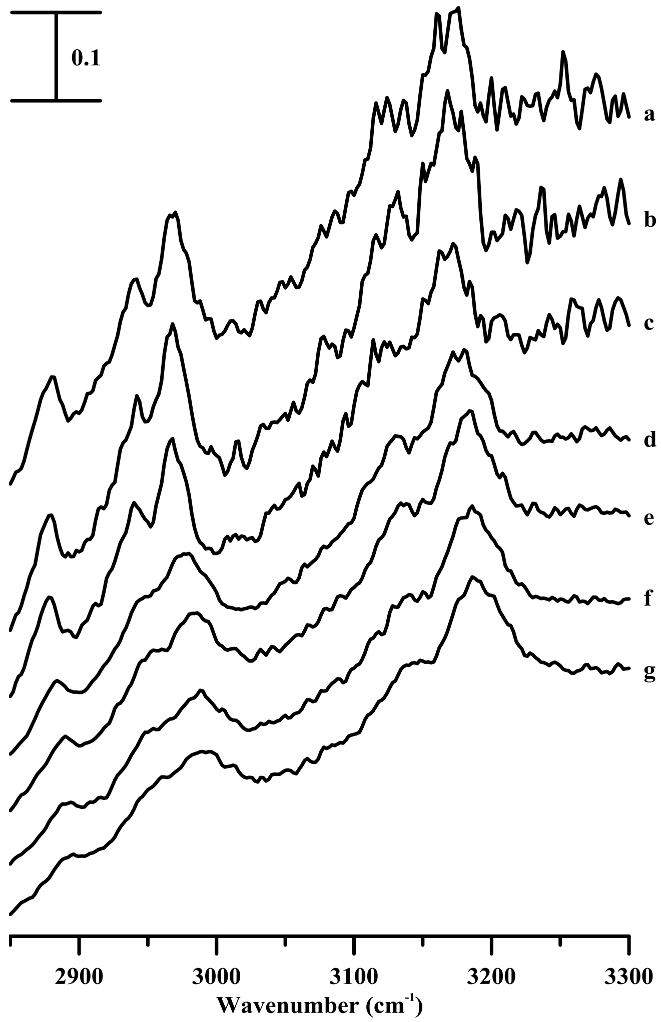

Figure S4. Infrared spectra of [C4C1Im][PF6] in a silica matrix obtained at (a) ambient pressure and at (b) 0.4, (c) 0.7, (d) 1.1, (e) 1.5, (f)1.8, and (g) 2.5 GPa

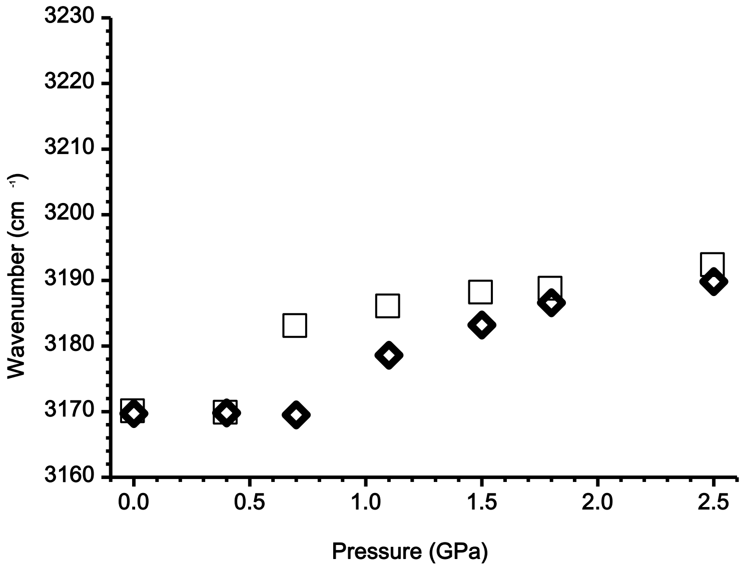

Figure S5. Pressure dependence of the C–H stretching frequencies at 3170 cm^{-1} of the pure [C4C1Im][PF6] (squares) and [C4C1Im][PF6] in a silica matrix (diamonds).
